# Using the MUSIC Inventory to Evaluate Pathology Courses: an Exploratory Study

**DOI:** 10.1007/s40670-022-01539-4

**Published:** 2022-04-06

**Authors:** Christopher Dimick Smith, Diane Kenwright, Tehmina Gladman

**Affiliations:** 1 Department of Pathology and Molecular Medicine, University of Otago Wellington, Wellington, New Zealand; 2 Education Unit, University of Otago Wellington, PO Box 7343, Newtown, Wellington 6242 New Zealand

**Keywords:** Motivation, Course evaluation, Medical education, Pathology education, MUSIC Inventory

## Abstract

**Background:**

The MUSIC Inventory evaluates student’s academic motivation across five constructs. We aimed to examine its use in undergraduate medical pathology courses.

**Activity:**

Students from three pathology courses completed questions for three factors of the MUSIC Inventory plus one open-ended question. We conducted an exploratory analysis of the survey data.

**Results:**

Results showed that the open-ended responses corresponded to differences in ratings on the MUSIC Inventory.

**Discussion:**

Combining an open-ended question with the MUSIC Inventory identified differences in student motivation plus aspects of each course that could be improved. The MUSIC Inventory is an appropriate evaluation method for pathology teaching.

## Background

One means of improving educational practice is by applying well-supported psychological research and theory, for example, motivation research. Academic motivation is motivation to persist in learning-related tasks [1]. Students lacking motivation put in less effort and learn less effectively [2]. Studies show that students’ academic motivation is related to feedback on quality and value of a course [3, 4]. The MUSIC model of academic motivation integrates several constructs to support teaching development [5]. The MUSIC model consists of five factors—empowerment, usefulness, success, interest, and caring.

Jones [6] developed an inventory based on the MUSIC model and, with colleagues, has investigated the validity of the MUSIC Inventory [7–10]. Research has found evidence for the validity of the MUSIC Inventory with students in professional programs including veterinary, pharmacy, medical, nursing, engineering, and offline and online health-related courses [11–16].

### Pathology at the Otago Medical School

University of Otago medical students study pathology as part of their medical education. In the last 3 years of the course, students are distributed among the Dunedin (DSM), Wellington (UOW), and Christchurch (UOC) campuses. At UOW, students attend face-to-face lectures and tutorials on anatomic pathology. Additionally, 4th year students are required to complete work on the online learning platform kuraCloud [17]. kuraCloud activities are optional for 5th years. kuraCloud is an online learning platform where teachers create interactive learning objects to support student self-directed learning, and students watch videos presented by a pathology lecturer, answer related questions, and complete case-based questions. Both year groups have optional online quizzes for formative assessment.

In 2019, in response to feedback and assessment results, the pathology course for DSM 4th-year students was altered to a blended learning format. Online, students completed the same kuraCloud work as UOW 4th years and participated in Wellington-based Zoom tutorials.

To evaluate the changes to the Dunedin 4th-year pathology course and compare it to the different ways in which Wellington 4th- and 5th-year pathology is taught, we explored the use of the MUSIC Inventory as a method for evaluating pathology learning. Our aim was to explore specific scales in the MUSIC Inventory as a viable method for evaluating course design. We asked two questions. First, would the MUSIC Inventory differentiate between the three groups’ ratings? Second, would students’ open-ended responses and related MUSIC factor ratings be consistent?

## Activity

Students from two campuses participated in this study: clinical 4th- and 5th-year students from UOW (UOW4, UOW5) and 4th-year students from DSM (DSM4). For the evaluation, students completed the MUSIC Inventory’s caring (6 questions), usefulness (5 questions), and success (4 questions) items on a six-point Likert-type scale [6]. Caring, usefulness, and success were chosen to reduce student burden and for their expected relevance in the evaluation of the pathology courses. Interest was considered less relevant for medical students [13, 18], and providing useful content and activities better reflected the aims of the pathology courses. Empowerment was also thought less relevant due to the highly structured nature of the medical curriculum.

In addition to the MUSIC questions, students completed a question about their expected grade in pathology, an open-ended question asking what helped and hindered learning, and demographics. From a practical perspective, the open-ended question was included to better understand specific aspects that contributed to students’ evaluation of the course. From a research perspective, if the MUSIC inventory is capturing important parts of a students’ evaluation of a course, it should align with students’ open-ended responses.

We administered the evaluation in October 2019 before end-of-year exams. Students were approached near the end of a pathology session and informed that the survey was optional but would be used to evaluate and improve the course. Students were provided a link to the Qualtrics survey and completed it using their own devices. After class, an email was sent encouraging non-respondents to participate.

Descriptive, between-group, and within-group analyses were conducted in SPSS. The data was initially tested for parametric assumptions of normality and homogeneity of variance, and appropriate non-parametric tests were used if needed. Bonferroni correction was applied to all post hoc tests. To explore the relationship between open-ended responses and MUSIC ratings, comments were coded to usefulness, success, caring, or ambiguous/none, and then as positive, negative, or neutral.

## Results

One hundred fifty students participated: 58 (39%) UOW4, 63 (42%) UOW5, and 29 (19%) DSM4 students. Females (62%) and New Zealand Europeans (51%) made up the majority of respondents (Table 1). Students’ responses to the usefulness, caring, and success items were generally positive (Table 2).

**Table 1:** Demographic data for the student sample

	UOW4	UOW5	DSM4
**Gender**			
Female	36	40	16
Male	18	21	12
Prefer not to answer	0	2	1
No response	4	0	0
**Ethnicity**			
New Zealand European	34	30	12
Māori	6	10	6
Chinese	4	11	4
Indian	2	7	2
Pacific Islander	0	1	1
Other	8	4	3
No response	4	0	1
**Age**			
Under 21	18	0	1
21–24	36	54	21
25 +	0	9	7
No response	4	0	0

**Table 2:** Descriptive statistics for the MUSIC factors by student group

Group	Sample	Usefulness		Caring		Success	
		Mean (SD)	Median	Mean (SD)	Median	Mean (SD)	Median
DSM4	29	3.75 (1.15)	3.80	4.36 (1.06)	4.67	3.72 (1.15)	4.00
UOW4	58	4.59 (0.69)	4.70	4.70 (0.68)	4.83	4.24 (0.64)	4.25
UOW5	62	4.42 (0.84)	4.60	5.06 (0.73)	5.00	3.98 (0.77)	4.00

Using a Kruskal–Wallis test, we examined the differences between groups for each scale (Table 3). DSM4 students provided significantly lower usefulness ratings than UOW4 and UOW5 students. UOW5 students gave higher caring ratings than UOW4 and DSM4 students. Finally, there were no significant differences between the groups for the success factor.

**Table 3:** Between-group comparison by scale and valence and within-group comparisons by group

Group comparisons by scale	Kruskal–Wallis test			Pairwise comparisons	
	*N*	*X*^2^(df)	*p*	*X*^2^ (std error)	*p*^a^
Usefulness	150	12.22(2)	0.002		
DSM4–UOW5				−26.90 (9.70)	0.017
DSM4–UOW4				− 33.84 (9.83)	0.002
UOW5–UOW4				6.94 (7.87)	1.00
Caring	150	14.66(2)	< 0.001		
DSM4–UOW5				−33.56 (9.73)	0.002
DSM4–UOW4				−10.91 (9.86)	0.806
UOW4–UOW5				−22.65 (7.89)	0.012
Success	150	4.13(2)	0.127		
DSM4–UOW5				−3.11 (9.66)	1.00
DSM4–UOW4				−16.60 (9.79)	0.269
UOW5–UOW4				13.50 (7.83)	0.254

Comparisons by valence of comments	Kruskal–Wallis test			Pairwise comparisons	
	*N*	*X*^2^(df)	*p*	*X*^2^ (sth error)	*p*^a^
Usefulness	67	14.80(2)	< 0.001		
Negative–neutral				13.25 (6.54)	0.129
Negative–positive				20.05 (5.34)	0.001
Neutral–positive				− 6.80 (6.97)	0.987

Within-subject comparisons by scale	Friedman’s two-way analysis of variance			Pairwise comparisons	
	*N*	*X*^2^(df)	*p*	*X*^2^ (sth error)	*p*^a^
DSM4	29	9.23(2)	0.010		
Success–usefulness				0.16 (0.26)	1.00
Success–caring				0.72 (0.26)	0.017
Usefulness–caring				−0.57 (0.26)	0.091
UOW4	58	15.94(2)	< 0.001		
Success–usefulness				0.56 (0.19)	0.008
Success–caring				0.68 (0.19)	0.001
Usefulness–caring				−0.12 (0.19)	1.00
UOW5	63	70.00(2)	< 0.001		
Success–usefulness				0.68 (0.18)	0.000
Success–caring				1.47 (0.18)	0.000
Usefulness–caring				−0.79 (0.18)	0.000

^a^Significance values have been adjusted using Bonferroni correction for multiple tests

We also examined differences in ratings within each group across the scales using Friedman’s test (Table 3). We found a significant within-subject effect for all three groups. All students showed significantly lower success ratings than caring ratings. There was no significant difference between caring and usefulness ratings for UOW4 students, while caring ratings were significantly higher than usefulness ratings for UOW5 students. Finally, for DSM4 students, there was no significant difference between usefulness and caring or success.

Eighty-two students answered the question: *Please comment on what/who helped or hindered your learning*. The most common help to learning was the provision of resources, with UOW5 also commending the pathology teachers and lectures. Negative responses varied by group. DSM4’s most common learning hindrances were related to Zoom and distance learning; for UOW4, it was related to perceived inconsistencies between the KuraCloud cases, class notes, and activities, while for UOW5, it was the organization of lecture content.

Sixty-seven comments were coded to usefulness, nine to success, eleven to caring, and three to ambiguous/none. As usefulness comments represented the majority, the valence of those responses was coded and analyzed. Students making only positive comments coded as positive, only negative comments coded as negative, and a mix of positive and negative comments coded as neutral.

Thirty-three usefulness comments were classified negative, 22 positive, and 12 neutral. A Kruskal–Wallis test found a significant group effect for valence. Post hoc tests revealed that usefulness ratings of students who made positive comments were significantly higher than those of students who made negative comments. There were no significant differences between the neutral and positive or negative groups (Table 3).

## Discussion

In this study, we explored the viability of the usefulness, caring, and success items of the MUSIC Inventory to evaluate pathology courses at the University of Otago. In line with our research questions, we found that the MUSIC Inventory could distinguish differences in student motivation between different courses. We also found a strong correlation between open-ended responses about usefulness and scores on the usefulness scale of the MUSIC inventory. We were able to use the open-ended questions in conjunction with the MUSIC Inventory scales to identify potential areas for improvement.

From the MUSIC Inventory, we found that the DSM4 pathology course students gave lower usefulness ratings than students in UOW4 and UOW5 courses. From the open-ended responses, DSM4 students found it more challenging to ask questions and get feedback from the instructor in the Zoom tutorials which may have contributed to the lower usefulness rating. As a result of this finding, we have made changes to the teaching for DSM to give them more opportunities to engage with tutors in person.

This result highlights an advantage of using the MUSIC Inventory. Both UOW4 and DSM4 students gave a similar proportion of positive and negative usefulness comments in their open-ended question. From this, we might conclude that although students differed in what they disliked about a course, their overall level of motivation was similar. However, the MUSIC Inventory enables a more refined understanding by clarifying the specific aspect of students’ motivation that was affected. This allowed us to interrogate the open-ended statements in more detail to determine exactly which aspect of usefulness students are missing.

We also found students tended to give lower ratings for success items. One plausible explanation for this finding is that 4th- and 5th-year students see patients as part of their training, and pathology may be a lower priority, leaving students feeling less confident about mastering pathology content in the time available. If this explanation is correct, it may help to remind students that although pathology content can be challenging, students have been successful despite increased workload.

We also investigated the relationship between open-ended responses and MUSIC Inventory ratings. Further supporting the validity of the MUSIC Inventory, students who provided positive comments tended to rate the course as more useful than students providing negative comments.

As this was an exploratory study, there are limitations. Although the content and activities of the Dunedin and Wellington pathology courses were broadly aligned, there were location-specific differences that could produce variations in MUSIC ratings. Additionally, due to the rotation timetable, we obtained a larger sample size in the Wellington pathology courses than the Dunedin pathology course which may form a source of variation.

Overall, our results suggest the MUSIC Inventory is a useful tool for evaluating pathology courses. It provides a more refined understanding of aspects of student motivation in a course, and when paired with open-ended questions can highlight areas of courses for improvement. Future research could focus on the specific factors of academic motivation that lead to improved student engagement. It may also be fruitful to study the MUSIC Inventory as a tool for curriculum development.

## Data Availability

The datasets generated and/or analyzed during the current study are available from the corresponding author on reasonable request.
